# Peripheral Intravenous Access in Preterm Neonates during Postnatal Stabilization: Feasibility and Safety

**DOI:** 10.3389/fped.2017.00171

**Published:** 2017-08-10

**Authors:** Nariae Baik-Schneditz, Gerhard Pichler, Bernhard Schwaberger, Lukas Mileder, Alexander Avian, Berndt Urlesberger

**Affiliations:** ^1^Division of Neonatology, Department of Paediatrics, Medical University of Graz, Graz, Styria, Austria; ^2^Research Unit for Neonatal Micro- and Macrocirculation, Department of Paediatrics, Medical University of Graz, Graz, Styria, Austria; ^3^Institute for Medical Informatics, Statistics and Documentation, Medical University of Graz, Graz, Styria, Austria

**Keywords:** preterm neonates, intravenous access, postnatal stabilization, arterial oxygen saturation, cerebral oxygenation

## Abstract

**Background:**

Current European Guideline for resuscitation recommends a centrally positioned umbilical venous catheter as the best option for administering necessary drugs. Especially in preterm infants, a frequently used alternative is the peripheral venous catheter.

**Methods:**

Two randomized controlled studies were conducted at the Division of Neonatology, Medical University of Graz. During neonatal resuscitation, a standardized protocol was filled out by an uninvolved observer including time points after birth of all attempts of venous puncture, time point of successful venous puncture, and total number of needed attempts. Arterial oxygen saturation (SpO_2_) and heart rate (HR) were measured using pulse oximetry at the right hand/wrist. In each neonate, either NIRO 200NX (Hamamatsu, Japan) or INVOS 5100C (Covidien-Medtronic, USA) were used to measure cerebral tissue oxygenation index (cTOI) and cerebral regional oxygen saturation (crSO_2_), respectively. SpO_2_, HR, and cTOI/crSO_2_ during and 1 min before and after successful venous punctures were analyzed.

**Results:**

70 protocols were reviewed. Data of 61 preterm neonates were analyzed. Mean gestational age was 31.5 ± 2.2 weeks, and the mean birth weight was 1,527 ± 541 g. In median, it needed one attempt [interquartile range (IQR) 1–2] to establish a peripheral venous catheter. In median, intravenous (IV) catheterization was successfully established 5 (IQR 4–9) min after birth. SpO_2_ and cTOI/crSO_2_ rose significantly following the percentiles during the first 10 min after the birth. HR did not change significantly.

**Conclusion:**

Peripheral IV catheterization during postnatal stabilization of preterm infants is feasible and successful in most of the cases at first attempt.

## Introduction

Gaining intravenous (IV) access is often needed within minutes after birth, especially in preterm neonates, for administering medications, fluids, and nutrients during postnatal stabilization (1). There are different methods for neonatal vascular access including umbilical venous, peripheral venous, peripherally inserted central and central venous catheters, or intraosseous lines. Current European Guideline for resuscitation and support of neonatal transition recommends a centrally positioned umbilical venous catheter (UVC) as the best option for administering necessary drugs during neonatal resuscitation (2). Especially in preterm infants, who often require ventilatory and/or cardio-circulatory support, umbilical venous catheterization may be challenging. A frequently used alternative is the peripheral venous catheter. In a recent multicentre observational study, the first-time success rate of peripheral IV line in neonatal intensive care unit (NICU) population was 45% (3). Until now, there are no data available on how many attempts are needed to insert a peripheral venous catheter successfully or the safety of placing a peripheral venous catheter and its effects on preterm neonates during stabilization immediately after birth.

The primary aim of the present study was to investigate how many attempts were needed to establish a peripheral IV catheter during postnatal stabilization. Secondary aims were (a) to determine the time needed to establish the peripheral venous catheter and (b) to investigate any potential side effects of the venous puncture on the vital parameters in preterm neonates.

## Materials and Methods

The present study is a retrospective analysis of exploratory parameters of two randomized controlled trials (4, 5) that were conducted at a tertiary center, the Division of Neonatology, Department of Paediatrics, Medical University of Graz, from April 2012 to December 2014. Studies were carried out in accordance with the recommendations of Regional Committee on Biomedical Research Ethics with written informed consent from the parents of neonates before birth. All parents gave written informed consent in accordance with the Declaration of Helsinki. The protocol was approved by the Regional Committee on Biomedical Research Ethics.

Peripheral venous catheterization was the primary vascular access during the study periods. Preterm infants with gestational age <34 weeks were included into the analysis, if there was an intention to treat with medication *via* IV catheter (Neoflon, Sweden) within the first 10 min after birth.

Arterial oxygen saturation (SpO_2_) and heart rate (HR) were measured with the IntelliVue MP30 Monitor (Philips, Netherlands) using pulse oximetry at preductal level on the right hand/wrist. In each neonate, either NIRO 200NX (Hamamatsu, Japan) or INVOS 5100C (Covidien-Medtronic, USA) were used to measure cerebral tissue oxygenation index (cTOI) and cerebral regional oxygen saturation (crSO_2_), respectively. The sensor was positioned on the forehead in each infant. The sensor on the forehead was secured with cohesive conforming bandage (Peha-haft, Harmann, Germany). All variables were stored continuously every second in the multichannel system alpha-trace digital MM (BEST Medical Systems, Austria) for subsequent analysis.

During neonatal resuscitation, a standardized protocol was filled out by an uninvolved observer including time points after birth of all attempts of venous puncture, time point of successful venous puncture, and total number of needed attempts. SpO_2_, HR, and cTOI/crSO_2_ during and 1 min before and after successful venous punctures were analyzed.

SPSS 23.0.0.2 (IBM Corp. 2015) was used for data analysis. A value of *p* < 0.05 was considered significant. Continuous variables are presented as mean ± SD or median and interquartile range (IQR), as appropriate. Categorical variables are presented as absolute and relative frequencies. Changes over time were evaluated using analysis of variance for repeated measurements. In cases when assumption of sphericity was violated as estimated by Mauchly’s test of sphericity, Greenhouse–Geisser correction was used.

## Results

Data of 61 of 70 preterm neonates were analyzed—9 neonates were excluded, because there was no intention to treat. They did not get IV catheterization during neonatal stabilization. Mean gestational age was 31.5 ± 2.2 weeks, and the mean birth weight was 1,527 ± 541 g. In median, it needed one attempt (IQR 1–2) to establish a peripheral venous catheter. In median, IV catheterization was successfully established 5 (IQR 4–9) min after birth, whereby in 38 (62.3%) preterm neonates, the peripheral venous catheter was established with the first attempt. All infants had monitoring of cerebral oxygenation (cTOI/crSO_2_ was measured in 48/23 neonates). All 61 preterm neonates received respiratory support *via* face mask, and 10 neonates needed intubation subsequently during postnatal stabilization.

Arterial oxygen saturation, HR, and cTOI/crSO_2_ before, during, and after the procedure are presented in Table 1. SpO_2_ (*p* < 0.001), cTOI (*p* = 0.001), and crSO_2_ (*p* = 0.001) rose significantly. HR increased too, but the rise did not reach significance (*p* = 0.199).

**Table 1:** Vital parameters before, during, and after intravenous (IV) puncture.

Vital parameters	Before IV puncture	During IV puncture	After IV puncture	*p*-Value
Arterial oxygen saturation (%)	75.8 ± 18.2	81.3 ± 14.0	84.0 ± 10.5	<0.001
Cerebral tissue oxygenation index (%)	56.7 ± 12.8	60.0 ± 11.6	61.7 ± 11.1	0.001
Cerebral regional oxygen saturation (%)	53.8 ± 24.1	64.1 ± 20.7	65.6 ± 19.8	0.001
HF (bpm)	146 ± 21	149 ± 22	150 ± 22	0.199

## Discussion

The present study demonstrates that peripheral venous catheterization during postnatal stabilization of preterm neonates is feasible, and in the majority of patients (62%), only one attempt was necessary. The number of first-time success rate in our study was even higher than the previously reported by Legemaat et al. in NICU population (3).

The recent European Guideline (2) suggests that “drugs are best given via a centrally positioned umbilical venous catheter.” However, according to the authors, it was not intended to exclude any technique merely by failing to mention it (6). Peripheral IV catheter is a feasible alternative, especially in preterm infants without need for extensive resuscitation, for instance, without need for chest compressions. Especially as UVC is not without complications: a recent study shows that up to 20.3% of neonates developed UVC-related complications (among these were malposition and remnants of catheter in the umbilicus) (7). In our present study, we demonstrated that a peripheral IV catheter can be inserted successfully with one attempt and within 5 min after birth. In the present study, SpO_2_ and cerebral regional oxygenation saturation, both rose, but changes were along the percentiles, which represent the physiological increase during the first 10 min after birth (8–10). Furthermore, HR showed a trend to rise, at least remained completely stable. The courses of these parameters suggest the absence of any disturbance of physiological adaptation during peripheral catheter insertion.

## Conclusion

Peripheral IV catheterization during postnatal stabilization of preterm infants is feasible and successful in most of the cases at first attempt. In addition, the IV puncture during postnatal stabilization did not affect the physiological neonatal transition concerning arterial and cerebral oxygenation and HR.

## Ethics Statement

This study was carried out in accordance with the recommendations of Regional Committee on Biomedical Research Ethics with written informed consent from all subjects. All subjects gave written informed consent in accordance with the Declaration of Helsinki. The protocol was approved by the Regional Committee on Biomedical Research Ethics.

## Author Contributions

Conception and design: BU, NB-S, and GP. Collection and assembly of data: NB-S, BS, LM, GP, and BU. Analysis and interpretation of the data; critical revision of the article for important intellectual content; final approval of the article: NB-S, BS, LM, GP, BU, and AA.

## Conflict of Interest Statement

The authors declare that the research was conducted in the absence of any commercial or financial relationships that could be construed as a potential conflict of interest.
